# Frictional Pressure Drop and Cost Savings for Graphene Nanoplatelets Nanofluids in Turbulent Flow Environments

**DOI:** 10.3390/nano11113094

**Published:** 2021-11-16

**Authors:** Reem Sabah Mohammad, Mohammed Suleman Aldlemy, Mu’ataz S. Al Hassan, Aziz Ibrahim Abdulla, Miklas Scholz, Zaher Mundher Yaseen

**Affiliations:** 1 Department of Business Administration, Faculty of Administration and Economic, University of Misan, Amarah 62001, Iraq; reem-sabah@uomisan.edu.iq; 2 Department of Mechanical Engineering, College of Mechanical Engineering Technology, Benghazi 11199, Libya; maldlemy@ceb.edu.ly; 3 Division of Advanced Nanomaterial Technologies, Scientific Research Center, Al-Ayen University, Nasiriyah 64001, Iraq; muataz@alayen.edu.iq; 4 Environmental Engineering Department, College of Engineering, Tikrit University, Tikrit 34001, Iraq; a.abdulla@tu.edu.iq; 5 Division of Water Resources Engineering, Faculty of Engineering, Lund University, 221 00 Lund, Sweden; 6 Department of Civil Engineering Science, School of Civil Engineering and the Built Environment, University of Johannesburg, Kingsway Campus, Johannesburg 2092, South Africa; 7 Department of Town Planning, Engineering Networks and Systems, South Ural State University (National Research University), 454080 Chelyabinsk, Russia; 8 Institute of Environmental Engineering, Wroclaw University of Environmental and Life Sciences, 50375 Wrocław, Poland; 9 New Era and Development in Civil Engineering Research Group, Scientific Research Center, Al-Ayen University, Nasiriyah 64001, Iraq; 10 College of Creative Design, Asia University, Taichung City, Taiwan

**Keywords:** graphene nanoplatelets, cost saving, power plant management, turbulent flow, pumping power, pressure drop

## Abstract

Covalent-functionalized graphene nanoplatelets (CF-GNPs) inside a circular heated-pipe and the subsequent pressure decrease loss within a fully developed turbulent flow were discussed in this research. Four samples of nanofluids were prepared and investigated in the ranges of 0.025 wt.%, 0.05 wt.%, 0.075 wt.%, and 0.1 wt.%. Different tools such as field emission scanning electron microscopy (FE-SEM), ultraviolet-visible-spectrophotometer (UV-visible), energy-dispersive X-ray spectroscopy (EDX), zeta potential, and nanoparticle sizing were used for the data preparation. The thermophysical properties of the working fluids were experimentally determined using the testing conditions established via computational fluid dynamic (CFD) simulations that had been designed to solve governing equations involving distilled water (DW) and nanofluidic flows. The average error between the numerical solution and the Blasius formula was ~4.85%. Relative to the DW, the pressure dropped by 27.80% for 0.025 wt.%, 35.69% for 0.05 wt.%, 41.61% for 0.075 wt.%, and 47.04% for 0.1 wt.%. Meanwhile, the pumping power increased by 3.8% for 0.025 wt.%, 5.3% for 0.05 wt.%, 6.6% for 0.075%, and 7.8% for 0.1 wt.%. The research findings on the cost analysis demonstrated that the daily electric costs were USD 214, 350, 416, 482, and 558 for DW of 0.025 wt.%, 0.05 wt.%, 0.075 wt.%, and 0.1 wt.%, respectively.

## 1. Introduction

### 1.1. Research Background

Nanofluids improve heating rates, reduce processing time, and extend the life of machinery, making them ideal for use in power, manufacturing, transportation, medical, microfluidics, and microelectronics [1,2]. Heat transfer efficiency is poor in engineering applications involving fluids, particularly when employing fluids such as engine oil (EO), ethylene glycol (EG), and water (DW), and research into alternatives is ongoing [3]. Nanofluids are fluids in which stable and homogeneous solid nanoparticles (metals, metal oxides, or carbon-based nanostructures) are suspended [4,5]. Nanoparticles (NPs) in the thermal boundary layer as well as their random movement within the fluid may have a positive impact on the convective heat transfer coefficient [6,7,8].

### 1.2. Research Motivation and Literature Review

Graphene is a breakthrough material due to its remarkable thermal, physical, and electrical properties [9,10]. Graphene is a single sheet of carbon (C) atoms organized in a hexagonal lattice arrangement [11]. Exfoliated graphene nanoplatelets (GNPs) are extracted from graphite layer by layer as the graphite collects stacks of graphene. The diameter of the graphene layers ranges from 1–100 µm, while their thickness is measured in nm [12]. The hydrophobic nanoparticles can be functionalized by covalent (functional groups) and non-covalent (surfactants) modification techniques [13,14,15]. The non-covalent approach depends on polar–polar interactions to prevent solid GNPs from sedimenting into a homogenous slurry by covering the graphene surface area with surfactants/polymers that act as stabilizers [16]. The presence and use of the aforementioned surfactants/stabilizers, on the other hand, compromises the purity of the GNPs and their subsequent qualities [17]. Binding with hydrophilic functional groups such as carbonyl, hydroxyl, carboxyl, sulfhydryl, amino, and phosphate groups is required for covalent functionalization [18].

The fully developed flow of GNPs-H_2_O nanofluid within a horizontally smooth, heated pipe was investigated experimentally and numerically at various mass fractions and heat flux intensities [19]. The pressure dropped by 9.1% for 0.025 wt.%, 10.2% for 0.05 wt.%, 13.1% for 0.075 wt.%, and 14.6% for 0.1 wt.%. Propylene glycol-coated graphene nanoplatelets suspended in water (PGGNP-Water) were experimentally tested [20]. Two heating rates, as 23,870 and 18,565 W/m^2^, were subjected to the test section, and the Reynolds number (Re) was in the range of 3900 ≤ Re ≤ 11,700. The maximum increase in the friction factor was computed from 4–14% at velocities of 1–3 m/s for a weight concentration of 0.1%. Yarmand et al. [21] produced GNPs-H_2_O nanofluid to study the heat transfer and thermodynamic properties in a square test section under a constant heat flux and turbulent flow conditions. Their results showed a 9.22% increment in the thermodynamics properties using 0.1 wt.% nanofluid at a Re of 17,500. Sadri et al. [6,7,8,9,10,11,12,13,14,15,16,17,18,19,20,21,22] produced stable and eco-friendly CGNPs-H_2_O nanofluids for heat transfer and hydrodynamic applications. The friction factor increased by ~3.79% for 0.1 wt.% They believed that the increased pressure drop was due to a minor rise in the viscosity of all of the CGNPs-DW nanofluids, which necessitated a nearly insignificant increase in the fluid velocity due to the constant Re. In experimental and simulation work, Abdelrazek et al. [23] explained the heat transfer and pressure drop of four nanofluids using two pipe configurations. They reported that the pressure dropped by 23% for Al_2_O_3_–DW, 24% for SiO_2_–DW, 29% for KRG, and 123% for GNP–SDBS.

### 1.3. Research Objectives

The use of nanofluids to improve convective heat transfer coefficients is common in the literature; nevertheless, additional research is needed to understand the negative side (pressure loss) of using carbon nanofluids in engineering applications [24]. Hence, this research aims to investigate pressure drops, pumping power consumption, and electric costs along heated pipes in fully developed turbulent flows, both experimentally and numerically. The characteristics of the CF-GNPs nanofluids were determined at 303K and were employed in the 3D-CFD model. A realizable (*k-ε*) model with enhanced wall treatment was used, with the Reynolds number range of 7500 ≤ Re ≤ 20,000.

## 2. Methodology

### 2.1. Preparation of Nanofluids

The pure nanomaterials (GNPs) were purchased from (VCN-Materials, Bushehr, Iran) and had the specifications of (carbon content = 95%, thickness = 32 nm, diameter = 5–20 μm, specific surface area = 150 m^2^/g). Chemicals and solvents such as pentaethylene glycol (PEG), aluminum chloride and hydrochloric acid (AlCl_3_/HCl), *N*, *N*-dimethylformamide (DMF), and tetrahydrofuran (THF) were obtained locally from (Sigma-Aldrich (M) SDN BHD, Sigma-Aldrich, Petaling Jaya, Selangor, Malaysia). The graphene nanoparticles (GNPs) were modified using covalent functionalization to introduce the carboxyl and hydroxyl functional groups (OH and COOH) [25]. The required GNP mass was weighed using a precision balance (OHAUS PA214, Parsippany, NJ, USA), and the nanofluid preparation was achieved using an Ultrasonic Processor (Vibra-Cell, Sonics, VC 750, 53 Church Hill Road, Newtown, PA, USA).

### 2.2. Characterization Techniques of Nanofluids

The UV-VIS absorbing profiles were taken from Perkin Elmer (Lambda 750, Shelton, CT, Waltham, MA, USA) device within the range of (190–3300 nm). The laser Doppler electrophoresis or phase analysis light scattering was conducted using Anton Paar (Litesizer 500, Graz, Austria) for the zeta potential and particle size testing. The T-degree of the aversion between near-NPs demonstrated a similar nanofluid dispersal load [26]. The SEM-VEGA3 tool from (Tescan, Brno, Czech Republic) was used to image the sample morphologies and to elementally analyze the covalent functionalized nanopowder of the CF-GNPs. The thermophysical properties of the working fluids at an inlet temperature of 303 K were obtained from previous studies [27] and were utilized in this work.

### 2.3. Numerical Parameters and Procedures

#### 2.3.1. Physical Model and Assumptions

**Figure 1:**
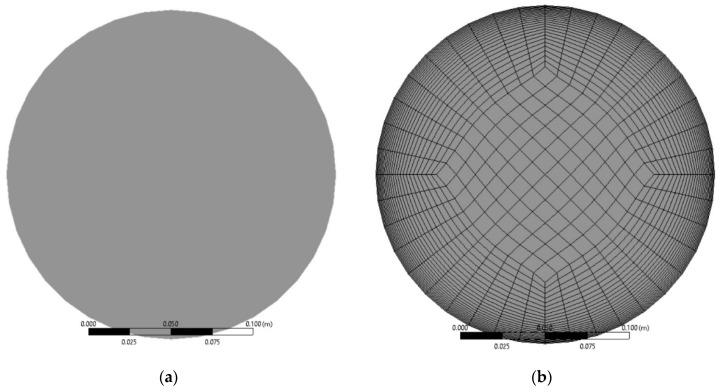
(**a**) Cross-section of the pipe flow and (**b**) grid of the computational domain.

Increasing the pressure drop of the CF-GNPs nanofluids along a circular heated-pipe was numerically solved using CFD under the condition of fully developed turbulent flows. The heated cross-section of the horizontal circular pipe was presented in Figure 1a. The total pipe length was 500 mm, the diameter was 20 mm, and the constant heat flux was 1000 W/m^2^. A grid was created using the meshing module of ANSYS-Fluent v2020R2. The physical domain modeled the fluid control volume and did not account for the tube wall thickness. Figure 1b presents the computational domain. Its 15 inflation layers were used to mesh the region adjacent to the walls, which were used to mesh the region adjacent to the walls due to significant velocity and temperature gradients.

Some assumptions were taken into account to solve the current model as follows [28]:

i. CF-GNPs/DW can be considered as a single-phase fluid.
ii. Base fluid (water) and CF-GNPs nanoparticles are within thermal equilibrium at zero relative velocity.
iii. The nanofluids are Newtonian fluids.
iv. Thermophysical properties of nanofluid samples change with increasing weight concentrations.
v. Its temperature dependence is negligible due to the minimal temperature variations throughout the study.

#### 2.3.2. Governing Equations

The conservation of mass, momentum, and energy for the single-phase efficient model are as follows [6,23]:

(1) $$\nabla .\left(\rho_{eff}\bar{V}\right)=0$$

(2) $$\nabla .\left(\rho_{eff}\bar{V}\bar{V}\right)=-\nabla \bar{P}+\mu_{eff}\nabla^{2}\bar{V}-\rho_{eff}\nabla .\left(\bar{vv}\right)$$

(3) $$\nabla .\left(\rho_{eff}Cp_{eff}\bar{V}\bar{T}\right)=\nabla .\left(\left(k_{eff}+k_{t}\right)\nabla \bar{T}\right)$$

where $\bar{V}$, $\bar{P}$, and $\bar{T}$ are the time-averaged flow variables, while $\bar{v}$ is the velocity fluctuation(s). The momentum equation $\rho_{eff}\nabla .\left(\bar{vv}\right)$ represents the turbulent shear stress, while $k_{eff}$ and $k_{t}$ are the effective molecular conductivity and the turbulent thermal conductivity, respectively.

Two additional equations must be used to solve the kinetic energy (*k*) and turbulent dissipation (*ε*). The coefficients were calculated using empirical methods and are only applicable to fully developed turbulent flows. Since the eddy viscosity can only be estimated using the turbulence length scale, the calculated turbulent diffusion happens on that scale. In reality, range(s) of motion all contribute to turbulent diffusion [29]. The gradient diffusion hypothesis is used in the (*k-ε*) model to correlate the Reynolds stresses, mean velocity gradients, and turbulent viscosity. In another context, the mentioned turbulent model performed poorly for the conjugated and complex flows, problems with the high-pressure gradient, the separation of the flows, and the strong streamline curvature. Its main flaws were its lack of sensitivity to pressure gradients and numerical stiffness when the equations were integrated via the viscous sublayer and treated with less-than-stable damping functions.

The (*k-ε*) turbulent model’s governing equation can be solved using the method outlined by Launder and Spalding [30]:

(4) $$\nabla .\left(\rho_{eff}kV\right)=\nabla .\left[\left(\frac{\mu_{t}}{\sigma_{k}}\right)\nabla \left(k\right)\right]+G_{k}-\rho_{eff}\varepsilon$$

(5) $$\nabla .\left(\rho_{eff}\varepsilon V\right)=\nabla .\left[\left(\frac{\mu_{t}}{\sigma_{k}}\right)\nabla \varepsilon \right]+\frac{\varepsilon }{k}\left(C_{1\varepsilon }G_{k}-C_{2\varepsilon }\rho_{eff}\varepsilon \right)$$

(6) $$G_{k}=\mu_{t}\left(\nabla V+{\left(\nabla V\right)}^{T}\right),\;\mu_{t}=\rho_{eff}C_{u}\frac{k^{2}}{\varepsilon }$$

*C_u_* = 0.09, *σ_k_* = 1, *σ_ε_* = 1.3, *C*_1*ε*_ = 1.44, *C*_2*ε*_ = 1.92 (7)

In this regard, (*μ_eff_*) is the effective viscosity of the nanofluid, while (*μ_t_*) is the viscosity coefficient in a turbulent regime.

The boundary conditions (BCs) for solving the CFD model’s governing equations are outlined in this section. The pipe wall was subjected to a constant heat flux (q_w_″) and no-slip (V_wall_ = 0) boundary conditions. The walls of the pipes were perfectly smooth, and its external surface was insulated. The working fluids (water and nanofluids) enter the heating pipe at a constant inlet temperature (T_in_ = 303 K) and had a uniform axial velocity (V_in_). Water and four samples of CF-GNPs in different wight concentrations were regarded as heat transfer fluids in this model. Flow assumed to be fully developed at the inlet of the pipe. Gravity was activated in the (Y^−^) direction and had the value of 9.81 m/s^2^. Moreover, the out-flow condition was imposed at the pipe outlet.

The finite volume method (FVM) was used to discretize partial differential equations (governing equations) into a set of linear algebraic equations, which made them numerically solvable. The second-order upwind scheme was used to discretize the convection and diffusion terms, and other appropriate variables appear in the governing equations. The velocity components were evaluated at the center of the control volume interfaces in staggered grid designs. All scalar quantities were estimated at the control volume’s center. The semi-implicit method for pressure-linked equations (SIMPLE) was used to link the pressure and velocity. ANSYS CFD uses a point implicit (Green–Gauss node-based gradient scheme) linear equation solver and an algebraic multigrid approach to solve the linear systems produced from the discretization schemes. The residual monitors had a convergence with an absolute criterion of < 10^−6^.

#### 2.3.3. Grid Optimization

Despite its known shortcomings, the (*k-ε*) turbulence model was used to solve the challenge, which included a poor performance in complex flows with high-pressure gradients, separation flow, and severe streamline curvature. The thin zone near a wall is its boundary layer, and the velocity gradient normal to the wall is significant. Laminar, transitional, and turbulent flows all have boundary layers. Turbulent flow shows laminar, sub-layer, and turbulence boundary layers, while laminar flow only has a laminar boundary layer. As per Table 1, the current geometry was treated, making it valid for (y^+^ < 5) [31]. The first cell height for a desired Y^+^ value can be calculated as follows:

(8) $$Re=\frac{\rho UD}{\mu }$$

(9) $$Y^{+}=\frac{\rho U_{\tau }\Delta y}{\mu }$$

(10) $$U_{\tau }=\sqrt{\frac{\tau_{w}}{\rho }}$$

(11) $$\tau_{w}=\frac{1}{2}\times C_{f}\times \rho \times U^{2}$$

*C_f_* = 0.079 × *Re*^−0.25^ (12)

where (*ρ*) and (*μ*) are the working fluid density and viscosity, respectively. (*U*) is the operating fluid velocity, (*D*) is the pipe diameter, and (*U_τ_*) is the friction velocity. Moreover, (*τ_w_*) and (*C_f_*) are the wall shear stress and skin friction factor, respectively.

#### 2.3.4. Simulations Validation and Verification

**Figure 2:**
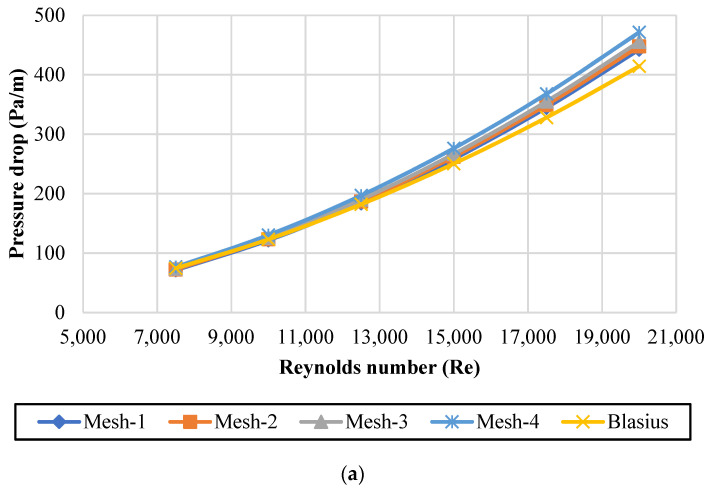

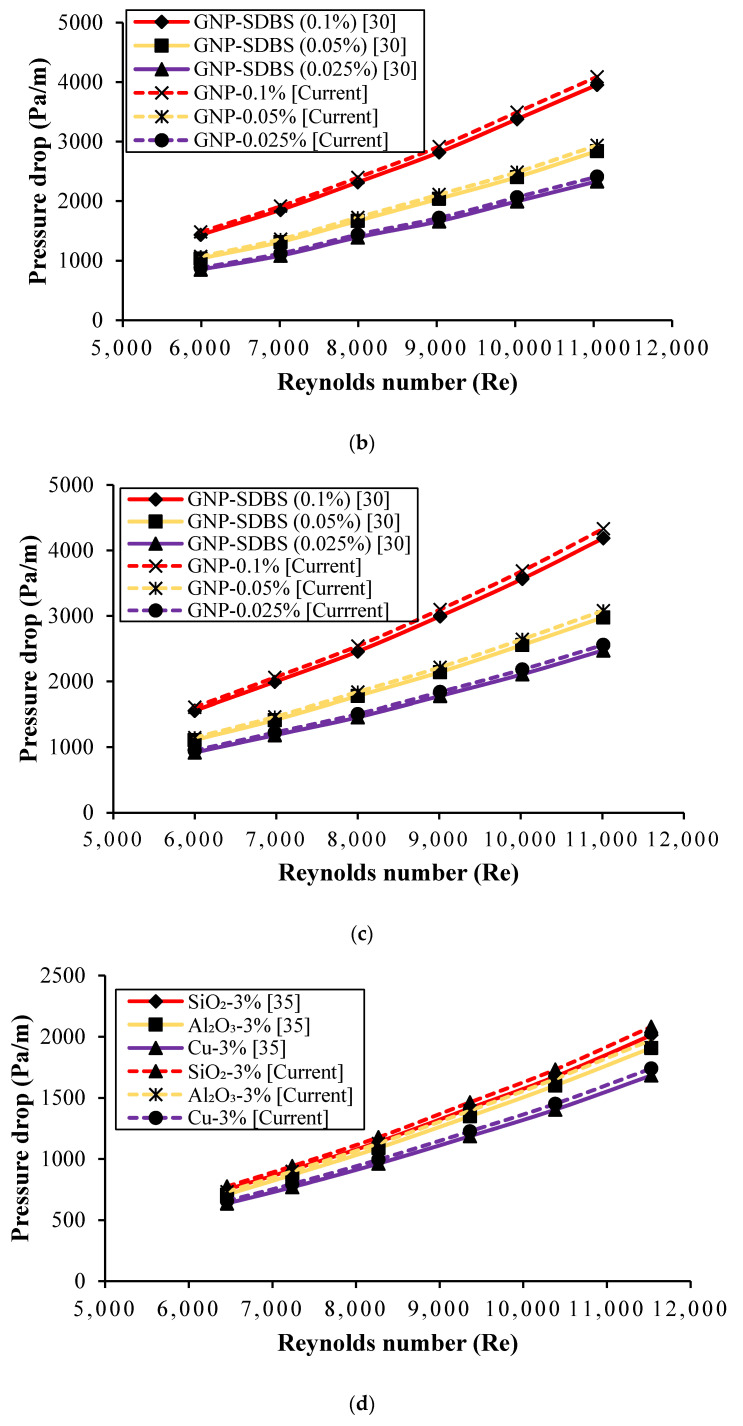
Validation and verification of current pressure drop data; (**a**) Comparison with the equation given by Blasius for DW; (**b**) comparison with GNPs nanofluids for square duct [23]; (**c**) comparison with GNPs nanofluids for circular duct [23]; (**d**) comparison with different nanofluids for circular pipe [28].

The pipe was 500 mm long and had a diameter of 20 mm. The working fluid entered the pipe tube at a fixed inlet temperature (*T_in_*) of 303K at a uniform axial velocity (*V_in_*) in the range of 7500 ≤ Re ≤ 20,000. For verification purposes, the obtained numerical results using four different grid computations such as (Grid-1 = 126,144 elements, Grid-2 = 177,536, Grid-3 = 219,146 and Grid-4 = 310,250) were compared against the theoretical data for conventional fluids. The pressure loss for the fully developed turbulent flow during the water run was compared with the Blasius correlation [32] (see Equation (13)). As per Figure 2a, the average errors between the simulations and Blasius correlation are 3.612% for Mesh-1, 4.114% for Mesh-2, 5.336% for Mesh-3, and 8.895% for Mesh-4. In the current study, Grid-1, with 126,144, was adopted due its accuracy, validity, and reliability. Moreover, as per Figure 2b,c, the pressure drop was calculated and compared with the experimental and numerical results of Abdel Razek et al. [23], who used GNPs nanofluids in square and circular ducts. The results were in good agreement, with an average deviation of 3.46% and 3.45%, respectively. Additionally, the variation of pressure drop per unit length with the Reynolds number was compared with the results of Abdel Razek et al. [28], who usedSiO_2_, Al_2_O_3,_ and Cu nanofluids in a heated pipe. As shown in Figure 2d, a similar trend was observed, with an average error of 3.44%.

(13) $$\frac{\Delta P}{L}=\frac{1}{2D}\times f\times \rho \times U^{2}$$

### 2.4. Cost Analysis

Using nanofluids in higher concentrations leads to a higher density and higher dynamic viscosity. The higher viscosity causes a higher pressure reduction, and high pumping power is required. In this sub-section, the required pumping power occurred when the nanofluids were driven into the thermal application. The pumping power in Watts can be estimated through the following formula: (${\dot{P}}_{Pump}=\frac{\dot{m}\times \Delta P}{\rho })$. To calculate the energy used in kilowatt-hours: ($kWh=P_{\left(W\right)}\times T_{\left(h/day\right)}÷1000_{\left(W\right)}$). Additionally, ($Price=Electricity_{\left(kWh\right)}\times Cost_{\left(price/kW\right)}$) can be used to calculate the electricity cost.

## 3. Applications Results and Analysis

### 3.1. Nanofluids Characterization and Thermophysical Characteristics

**Figure 3:**
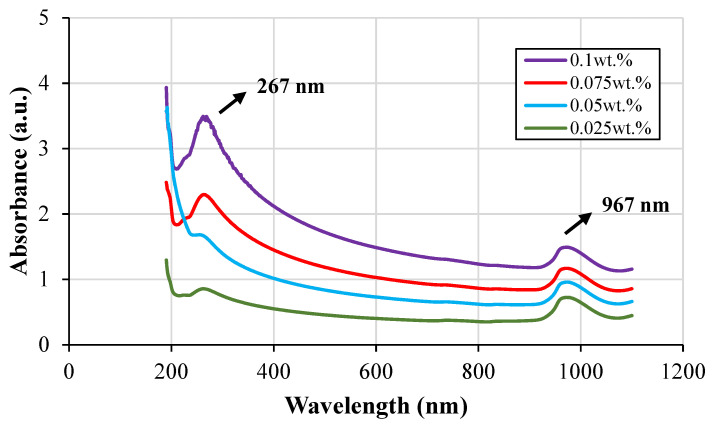
UV-Visible spectroscopy of CF-GNPs-H_2_O nanofluids with various nanoparticle concentrations.

Figure 3 presents the UV–Vis spectrum for CF-GNPs-H_2_O nanofluids in different mass fractions. There is a maximum peak at the absorption range of ~260–270 nm for all of the tested samples. The maximum and minimum absorption peaks are located at ~267 nm and ~967 nm, respectively. They can be credited to the π→π* transitions of the C=C bonds [33]. As per the Beer–Lambert law of absorbance, the peak intensity is directly related to the mass concentration of CF-GNPs [34].

**Figure 4:**
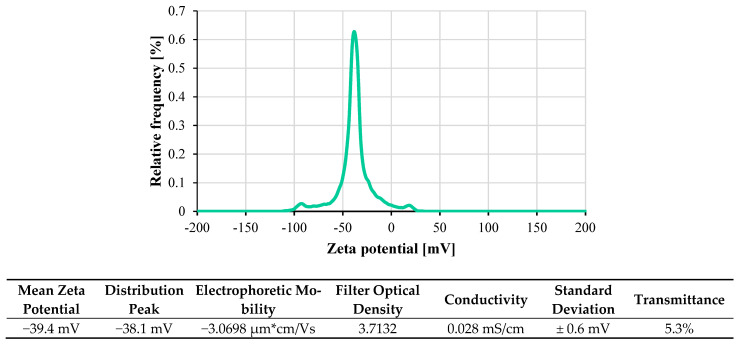
The distribution of the zeta capacity on the CF-GNPs nanofluid at 25 °C.

**Figure 5:**
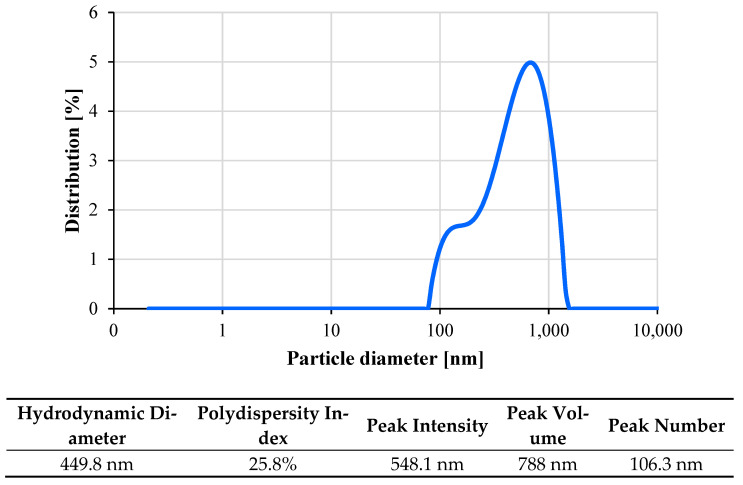
Particle size distributions for GNPs nanofluid at 25 °C.

Figure 4 shows the polydispersity index (PDI) and zeta potential for the CF-GNPs at natural pH values. Both values represent the electrostatic interactions between the nano-colloidal particles, which can quantify the dispersion’s homogeneity and stability [35]. At 25 °C and after being sonicated for 1 hr, the zeta potential becomes more negatively charged at ~−39.4 mV. The dynamic light scattering (DLS) approach was used in an aqueous solution to determine the size distribution and the average size of the produced graphene nanoparticles suspended in water. As per Figure 5, the average size of the GNPs was 548.1 nm, and its size distribution was within 77.4–1550.5 nm. The low PDI of 0.258 suggests a single and uniform particle size distribution in the solution.

**Figure 6:**
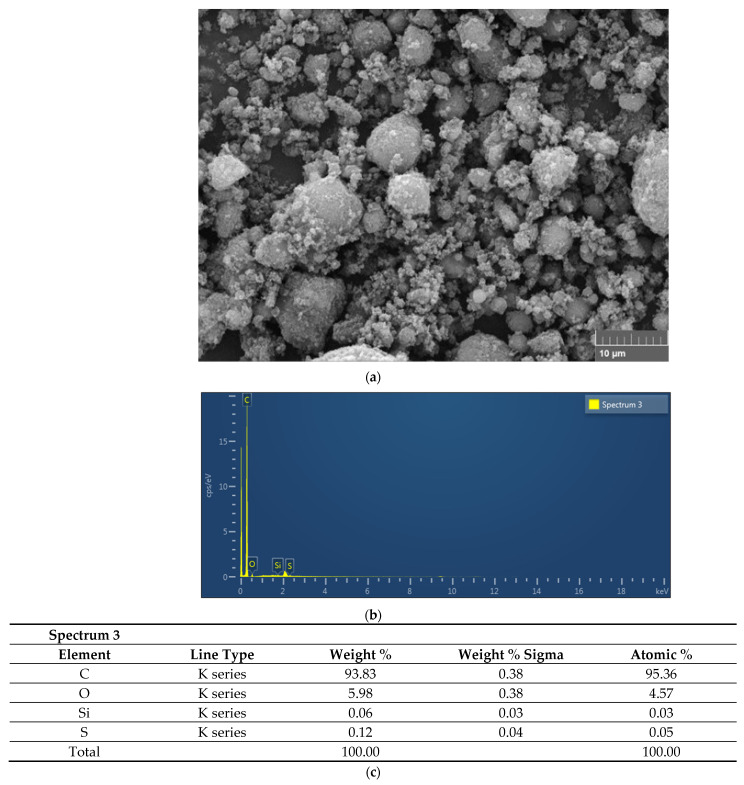

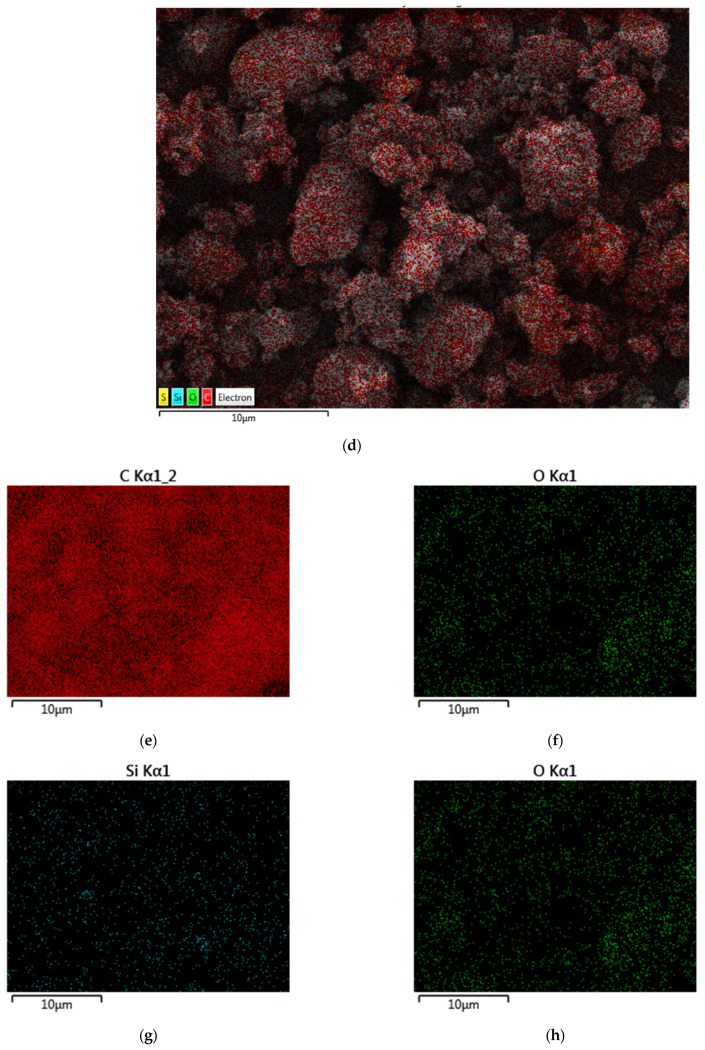
SEM and EDX mapping examination of CF-GNPs; (**a**) SEM image, (**b**) EDX mapping analysis, (**c**) EDX elemental analysis, (**d**) EDS layered image; (**e**) carbon (C) mapping; (**f**) oxygen (O) mapping; (**g**) silicon (Si) mapping; and (**h**) sulfur (S) mapping.

The dispersion and stabilization of the GNPs-nanofluid were imaged using an SEM. It can be seen in Figure 6 that the GNPs lack aggregation and are well dispersed. A highly wrinkled structure is also evident, which can be attributed to the functionalization via a strongly acidic medium. Additionally, Figure 6 shows the elements reported by the EDX measurements of the GNPs; carbon (C), oxygen (O), silicon (Si), and sulfur (S), with the corresponding atomic content being 95.36%, 4.57%, 0.03%, and 0.05%, respectively. These values confirm the excellent quality of the tested samples and agree with the results found in the literature [36].

**Figure 7:**
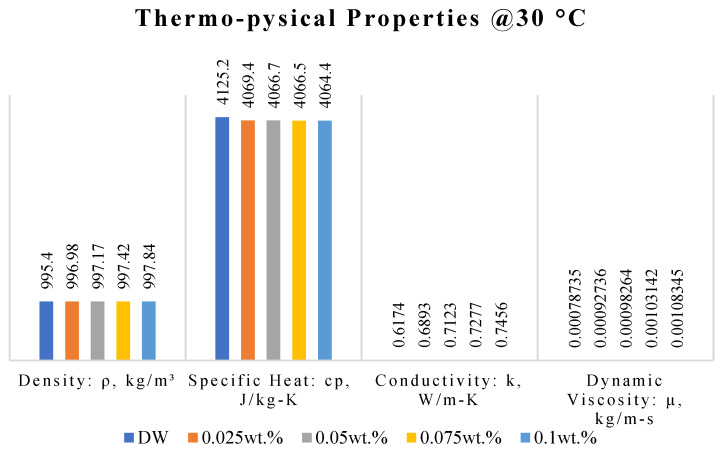
Thermo-physical properties of base fluid and nanofluids with different mass fractions at 30 °C.

Figure 7 depicts the thermo-physical properties of DW and nanofluids with four different mass fractions at the bulk temperature of 30 °C. The density and specific heat capacity of the nanofluids did not increase or decrease significantly. Meanwhile, the thermal conductivity and dynamic viscosity increased by 11.646% and 17.782%, 15.371% and 24.803%, 17.865% and 30.999%, and 20.764% and 37.607%, respectively, for 0.025%, 0.05%, 0.075% and 0.1%. The increase in the thermal conductivity can be attributed to the random Brownian motion of nanomaterials in an aqueous solution as well as the overwhelming high thermal conductivity supplied by graphene [37]. The viscosity of the nanofluids increases with increasing mass fraction, which is consistent with the previous results [38]. The cause for this can be loosely stated as follows: the solid particles remain stable in the system, resulting in an increase in the shear stress with the water molecules in the base liquid, which increases the viscosity.

### 3.2. Frictional Pressure Drop and Nanofluid Flow

**Figure 8:**
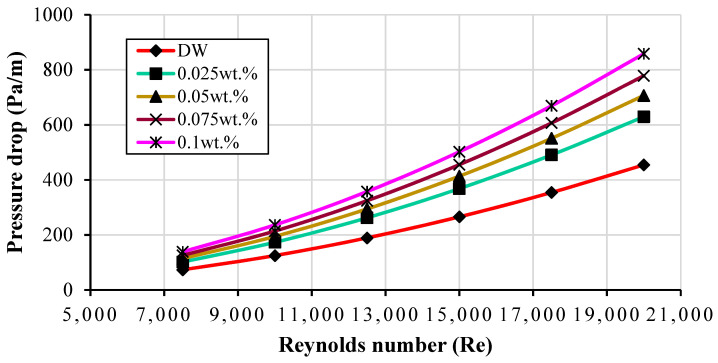
The frictional pressure loss of DW and different mass fractions of CF-GNPs versus different Reynolds numbers.

The flow properties of CF-GNPs must be determined to establish their application. The simulation seemed to confirm that the pressure drop relies upon the mass concentration of CF-GNPs and flow velocity (see Figure 8). The viscous drag effects of nanofluids increase the pressure drop (Equation (13)), where the friction factor (main variable of pressure drop) is mainly influenced by the density of the graphene nanofluids changing due to the increase in the mass concentration of the CF-GNPs. A significant parameter that increases the friction factor and pressure drop of the nanofluids is the density of the CF-GNPs [19]. Relative to the DW, the frictional pressure drop increased by 27.80% for 0.025 wt.%, 35.69% for 0.05 wt.%, 41.61% for 0.075 wt.%, and 47.04% for 0.1 wt.%, respectively, which can be attributed to the momentum diffusivity of the many types of circulating fluids [23]. The pressure loss in the flow regime can be directly linked to the fluid’s viscosity, where the latter increases the pumping power (detrimental). When designing heat exchangers, heat transfer and pumping power are critical (need to be minimized), as both variables significantly affect the evaluation of the nanofluid performance in thermal applications. Pumping power is the main cause of a fully developed turbulent condition in a circular tube that has been subjected to a uniform heat wall flux via $\left(\frac{\dot{W}}{{\dot{W}}_{DW}}\right)={\left(\frac{\mu }{\mu_{DW}}\right)}^{0.25}{\left(\frac{\rho }{\rho_{DW}}\right)}^{2}$ [39]. The formula seems to signify that the pumping power has a directly proportional relationship with the CF-GNPs nanoparticle concentrations of 3.8%, 5.3%, 6.6%, and 7.8% for 0.025 wt.%, 0.05 wt.%, 0.075 wt.%, and 0.1%-wt., respectively. The increase in the frictional pressure drop can be compared to those reported in the literature involving carbon nanomaterials within a heated pipe, as per Table 2. Figures S1 and S2 in Supplementary Materials display the contours of the temperature and velocity at different cross-sections (planes). The range of colors (blue to red) represents the temperature and velocity profiles (minimum to maximum) within the pipe.

### 3.3. Pumping Power and Cost Savings

**Figure 9:**
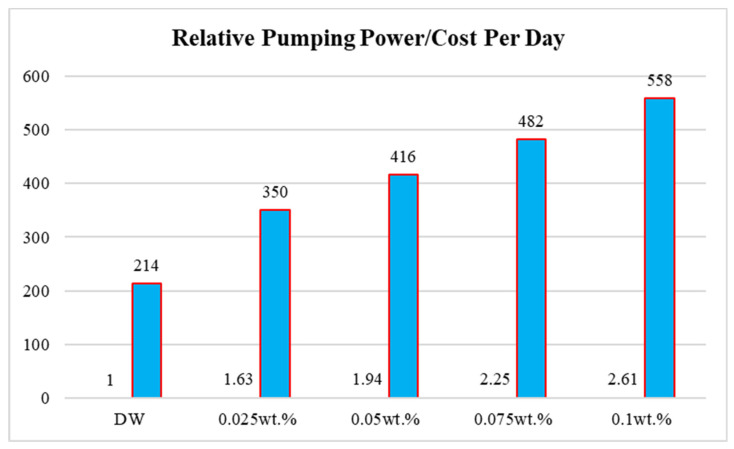
Relative pumping power and the electricity cost per day using base fluids and nanofluids in different mass percentages.

In this subsection, the pumping power and cost savings were discussed and assessed. As per Figure 9, the system consumed more power due the use of nanofluids over base fluids, achieving consumption rates of 1.63, 1.94, 2.25, and 2.61 for 0.025 wt.%, 0.05 wt.%, 0.075 wt.%, and 0.1 wt.%, respectively. This was calculated by using the formulas to calculate the energy consumption costs. Moreover, the electricity cost was estimated for use standard use on a daily basis (8 h.), and the nanofluids showed a higher cost due the higher pumping power consumption that is necessary. The price per kWh was determined to be USD1.2, and daily electric cost was calculated as USD 214, USD 350, USD 416, USD 482, and USD 558 for DW, 0.025 wt.%, 0.05 wt.%, 0.075 wt.%, and 0.1 wt.%, respectively.

## 4. Conclusions

This research described the experimental and numerical methods used to determine the frictional pressure drop in a smooth, heated pipe employing CF-GNPs-H_2_O nanofluids as heat transfer fluids. The experimental approach involved preparing the CF-GNPs and their characterization via UV-Vis, zeta potential, nanoparticle size distribution, and SEM-EDX, while numerical analyses involved a 3D-CFD approach using a fully developed turbulent flow test section of a circular heated pipe. Compared to the Blasius formula, the model established in this study was validated and reported an average deviation of 4.849%. Relative to the DW, the frictional pressure drop increased by 27.80% for 0.025 wt.%, 35.69% for 0.05 wt.%, 41.61% for 0.075 wt.%, and 47.04% for 0.1 wt.%, respectively. Higher pumping power was required due to the reduction in pressure drop relative to the base fluid as 1.63, 1.94, 2.25, and 2.61 for 0.025 wt.%, 0.05 wt.%, 0.075 wt.%, and 0.1 wt.%, respectively. In this regard, the additional daily electrical cost was as USD 214, USD 350, USD 416, USD 482, and USD 558 for DW 0.025 wt.%, 0.05 wt.%, 0.075 wt.%, and 0.1 wt.%, respectively.

## Figures and Tables

**Table 1:** Grid optimization with improving Y^+^.

CFD Simulation	Δ*y*	*Y* ^+^
1	2.89 × 10^−5^	1.790
2	2.25 × 10^−5^	1.571
3	1.85 × 10^−5^	1.433
4	1.58 × 10^−5^	1.339
5	1.38 × 10^−5^	1.269

**Table 2:** Experimental and numerical studies on the use of carbon nanostructured nanofluids in heated pipe.

Ref.	Study Type	Basefluid	Nanoparticles		Flow Type	Remarks
			Type	Concentration		
[10]	Exp.	H_2_O	RGO, RGO+CNT, RGO+CNF, RGO+GNPs	0.05 wt.%	Turbulent	The results recorded a small increase in pressure loss with respect to H_2_O.
[11]	CFD	H_2_O	CGNPs	0.025–0.1 wt.%	Turbulent	A slight increase in the pressure loss for CGNPs compared with those for DI water.
[24]	Exp., CFD	H_2_O	GNPs	0.025–0.1 wt.%	Turbulent	The increase in the pressure drop was in the range 0.4%-14.6%.
[25]	Exp.	H_2_O	PGGNP	0.025–0.1 wt.%	Turbulent	The highest increment in friction factor was calculated from 4% to 14% at velocities from 1 to 3 m/s using 0.1wt.%.
[26]	Exp.	H_2_O	f-GNP	0.02–0.1 wt.%	Turbulent	Friction factor increased by 9.22% using 0.1wt.% at Re of 17,500.
[27]	Exp.	H_2_O	CGNPs	0.025, 0.075, 0.1 wt.%	Turbulent	The friction factor increased by about 3.79% using 0.1 wt.%.
[28]	Exp., CFD	H_2_O	GNPs, KRG, Al_2_O_3_, SiO_2_	0.025–0.01wt.%	Turbulent	The pressure drop increased about 23%, 24%, 29%, and 123% for Al_2_O_3_–DW, SiO_2_–DW, KRG, and GNP–SDBS, respectively.
[40]	Exp.	Pure oil	GNPs, MWCNTs	0.05wt.%, 0.1wt.%, 0.2wt%, 0.5wt.%	Laminar	The highest-pressure drop was observed in the presence of OA-MWCNTs in pure oil at a concentration of 0.5 wt.% and 50 mL/s, which was 6.3%.
[41]	Exp., CFD	H_2_O	GNPs, TiO_2_	0.5, 0.75, and 1wt.%	Laminar-Turbulent	The maximum pressure drop was 1.2 times relative to DW at the highest Re for 1wt.% GNPs.
**Current study**	**CFD**	**H_2_O**	**CF-GNPs**	**0.025–0.1 wt.%**	**Turbulent**	**Pressure drop increased by 27.80%, 35.69%, 41.61% and 47.04%, respectively.**

## Data Availability

Data are presented in the article.

## Supplementary Materials

The following are available online at https://www.mdpi.com/article/10.3390/nano11113094/s1. Figure S1: Temperature contours of DW and different mass fractions of CF-GNPs at Re = 7500 and different cross-sections. Figure S2: Velocity contours of DW and different mass fractions of CF-GNPs at Re = 7500 and different cross-sections.

## Nomenclature

Al_2_O_3_	Aluminum oxide
AlCl_3_	Aluminum chloride
CFD	Computational fluid dynamics
CF-GNPs	Covalent functionalized Graphene nanoplatelets
D	Pipe diameter
DLS	Dynamic light scattering
DMF	N, N-dimethylformamide
DW	Distilled water
EDX	Energy-dispersive X-ray spectroscopy
EG	Ethylene glycol
EO	Engine oil
FE-SEM	Scanning electron microscopy
HCl	Hydrochloric acid
K_eff_	Effective molecular conductivity
PDI	Polydispersity index
PEG	Pentaethylene glycol
PGGNP	Propylene glycol-treated graphene Nanoplatelet
Re	Reynolds number
SDBS	Sodium dodecylbenzene sulfonate
SiO_2_	Silicon dioxide
THF	Tetrahydrofuran
T_in_	Inlet temperature
U	Freestream velocity
U_eff_	Effective viscosity of nanofluid
UV-visible	Ultraviolet-Visible—Spectrophotometer
V_in_	Inlet velocity
V_wall_	Wall velocity
$\dot{W}$	Hydraulic pumping power (W)
Y+	Dimensionless wall distance
ΔP	Pressure drop (Pa/m)
Δy	Wall spacing

## Greek Symbols

*ε*	Turbulent dissipation
*ρ*	Density
*τ_w_*	Wall shear stress
*µ*	Viscosity
*C_f_*	Skin friction factor
*k*	Kinetic energy
*μ_t_*	Coefficient of viscosity in turbulent Regime
*U_τ_*	Friction velocity
*k_t_*	Turbulent thermal conductivity
*f*	Friction factor
