# Supplementary material for: Frictional Pressure Drop and Cost Savings for Graphene Nanoplatelets Nanofluids in Turbulent Flow Environments
Source: Nanomaterials (Basel). 2021 Nov 16;11(11):3094. doi: 10.3390/nano11113094 (PMC8620931; doi:10.3390/nano11113094)
Supplement: Supplementary file 1 [file nanomaterials-11-03094-s001.zip › nanomaterials-1435867-supplementary.pdf]

Supplementary Materials

Frictional Pressure Drop and Cost Savings for Graphene Nanoplatelets Nanofluids in Turbulent Flow Environments

Reem Sabah Mohammad [1], Mohammed Suleman Aldlemy [2], Mu'ataz S. Al Hassan [3], Aziz Ibrahim Abdulla [4], Miklas Scholz [5,6,7,8,*] and Zaher Mundher Yaseen [9,10,*]

[1] Department of Business Administration, Faculty of Administration and Economic, University of Misan, Amarah 62001, Iraq; reem-sabah@uomisan.edu.iq
[2] Department of Mechanical Engineering, College of Mechanical Engineering Technology, Benghazi 11199, Libya; maldlemy@ceb.edu.ly
[3] Division of Advanced Nanomaterial Technologies, Scientific Research Center, Al-Ayen University, Nasiriyah 64001, Iraq; muataz@alayen.edu.iq
[4] Environmental Engineering Department, College of Engineering, Tikrit University, 34001, Iraq; a.abdulla@tu.edu.iq
[5] Division of Water Resources Engineering, Faculty of Engineering, Lund University, Lund 221 00, Sweden
[6] School of Civil Engineering and the Built Environment, Department of Civil Engineering Science, University of Johannesburg, Kingsway Campus, Johannesburg 2092, South Africa
[7] Department of Town Planning, Engineering Networks and Systems, South Ural State University (National Research University), Chelyabinsk 454080 , Russia
[8] Institute of Environmental Engineering, Wroclaw University of Environmental and Life Sciences, Wrocław 50375, Poland
[9] New era and development in civil engineering research group, Scientific Research Center, Al-Ayen University, Nasiriyah 64001, Iraq
[10] College of Creative Design, Asia University, Taichung City, Taiwan
* Correspondence: miklas.scholz@tvrl.lth.se (M.S.); yaseen@alayen.edu.iq (Z.M.Y.)

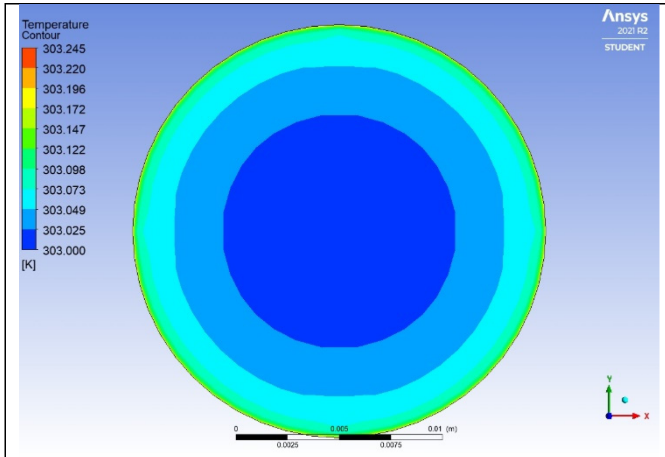

DW@ x/D = 10

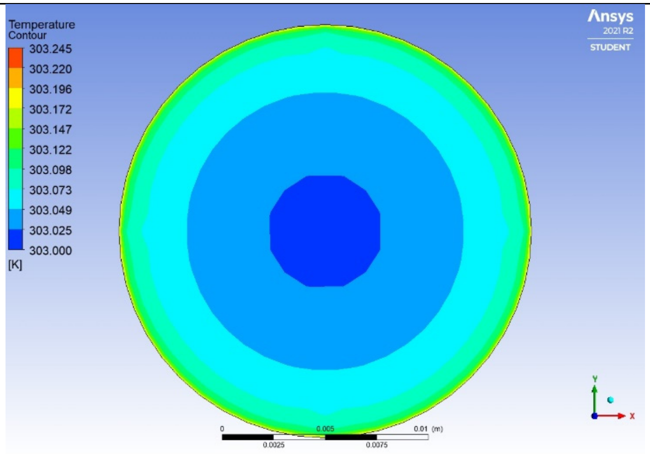

DW@ x/D = 15

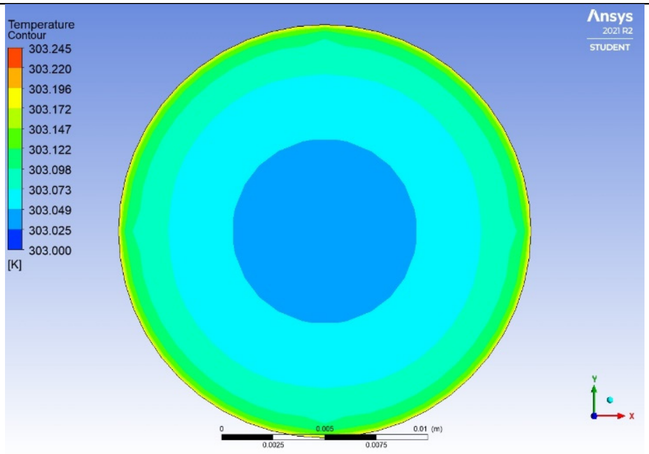

DW@ x/D = 20

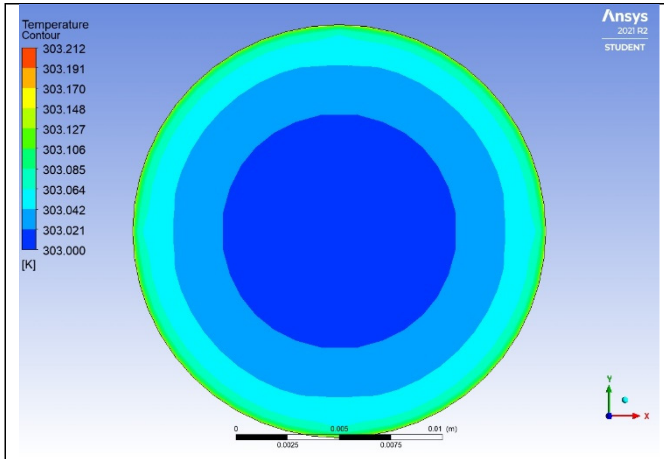

0.025%@ x/D = 10

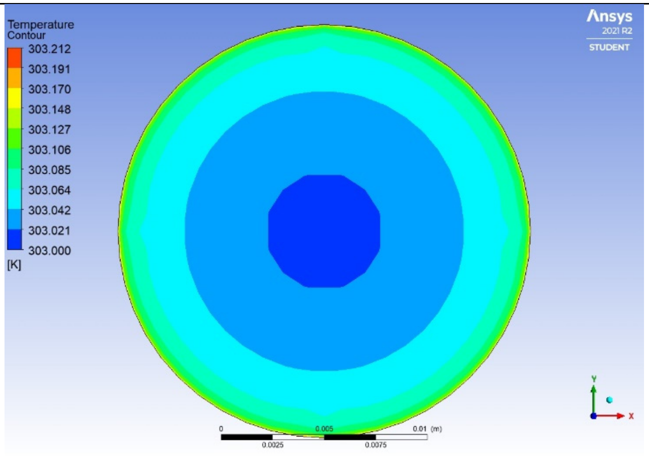

0.025%@ x/D = 15

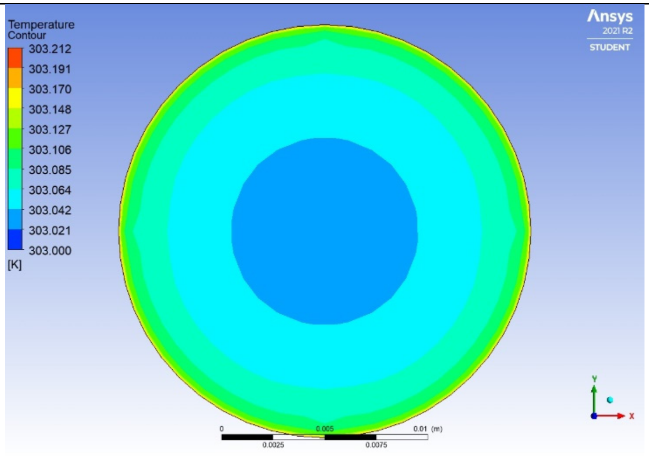

0.025%@ x/D = 20

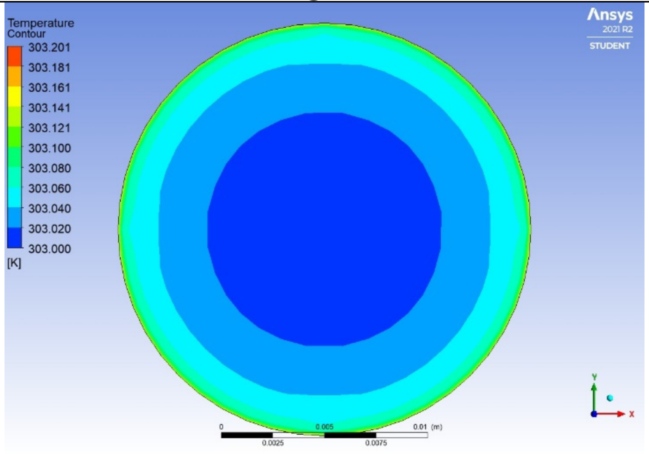

0.05%@ x/D = 10

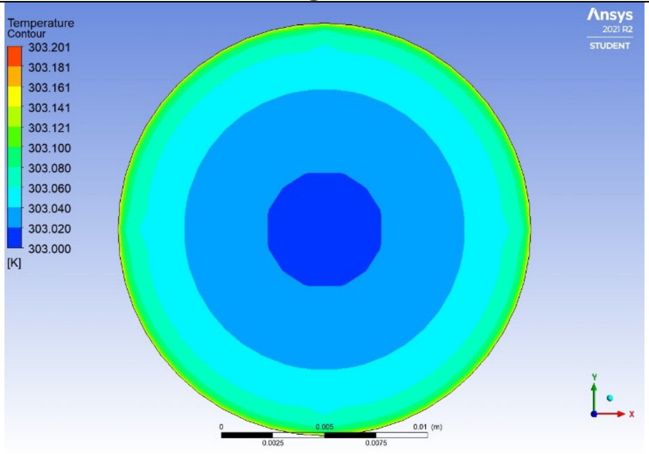

0.05%@ x/D = 15

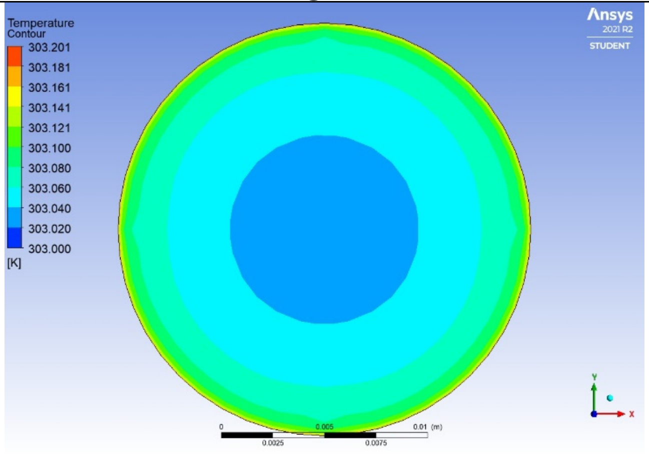

0.05%@ x/D = 20

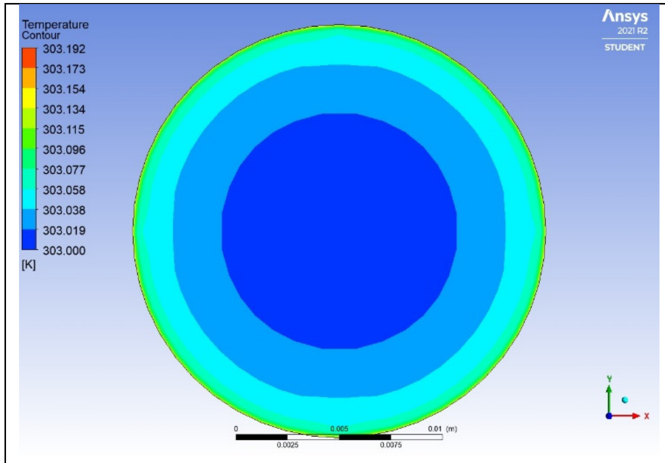

0.075%@ x/D = 10

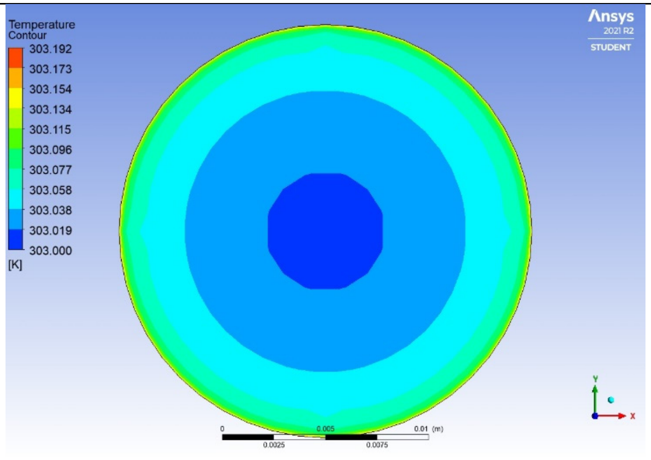

0.075%@ x/D = 15

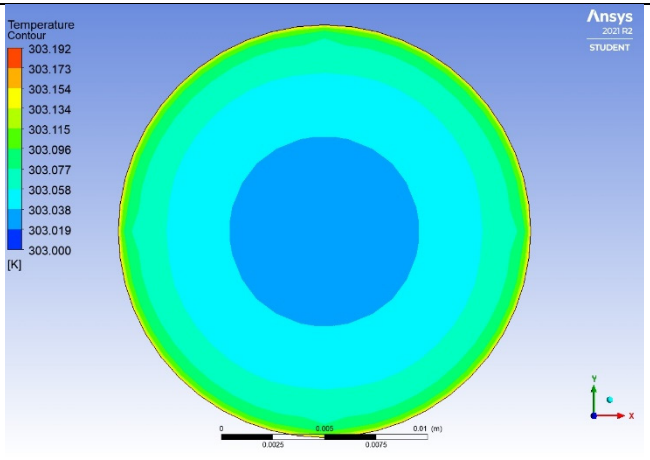

0.075%@ x/D = 20

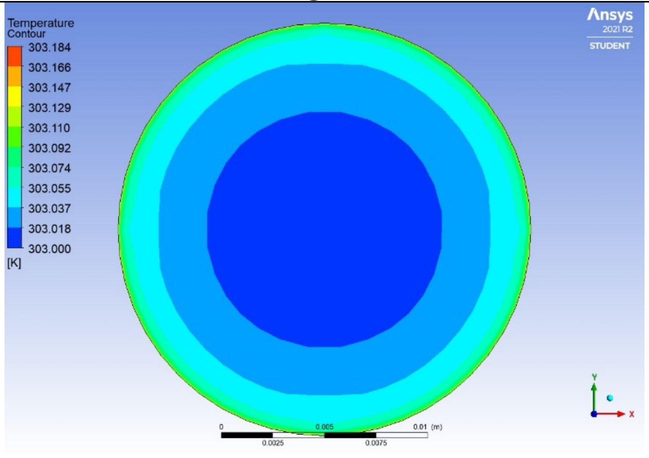

0.1%@ x/D = 10

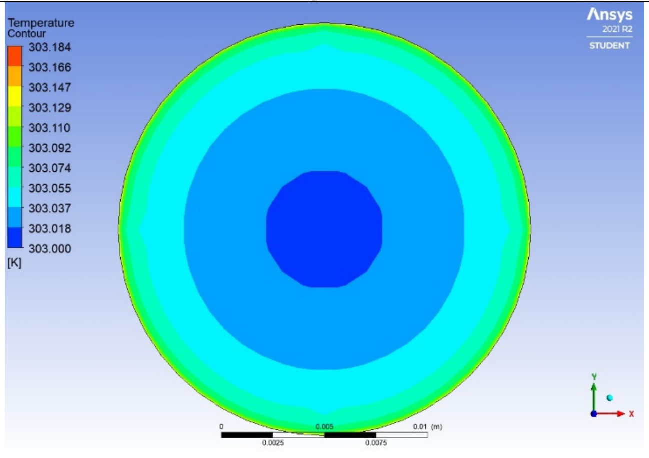

0.1%@ x/D = 15

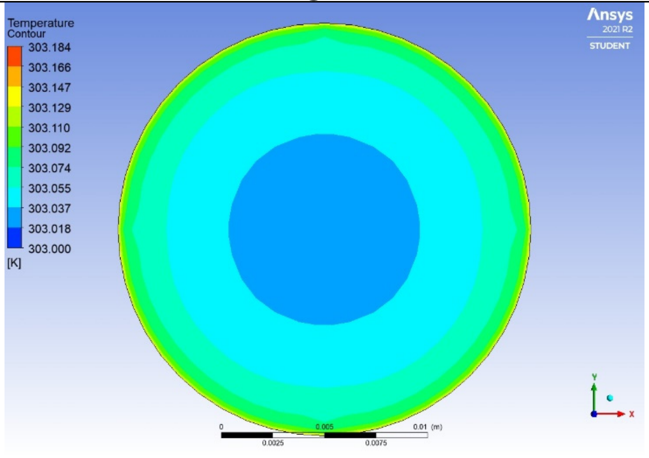

0.1%@ x/D = 20

Figure S1: Temperature contours of DW and different mass fractions of CF-GNPs at Re = 7,500 and different cross-sections.

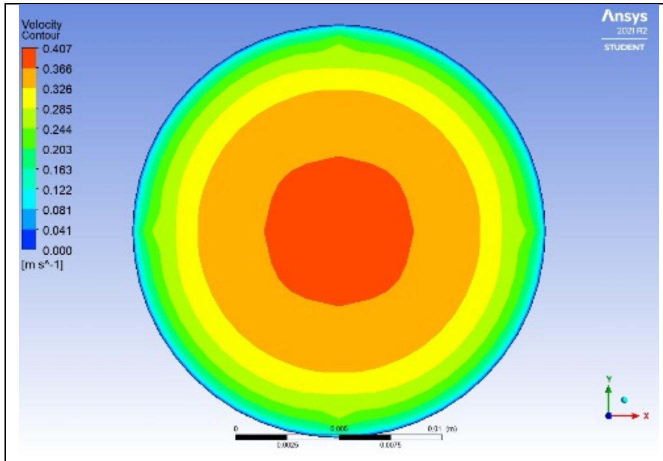

DW@ x/D = 5

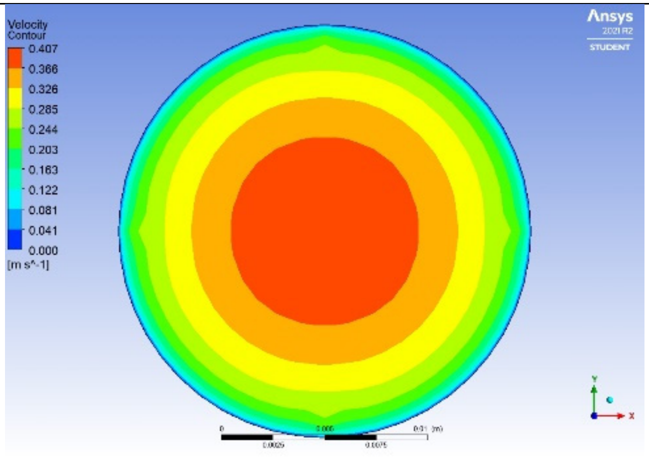

DW@ x/D = 10

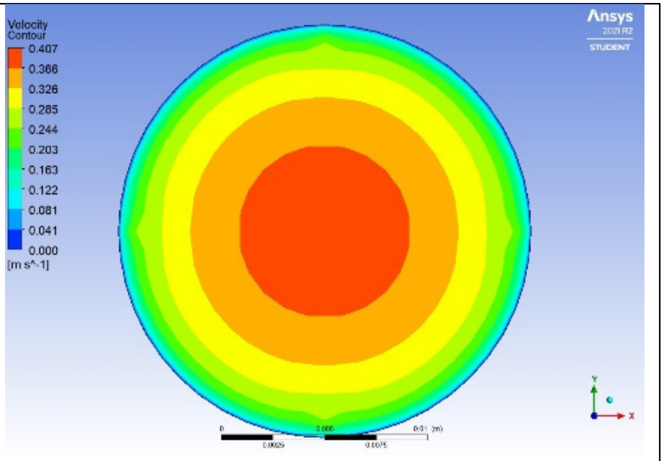

DW@ x/D = 15

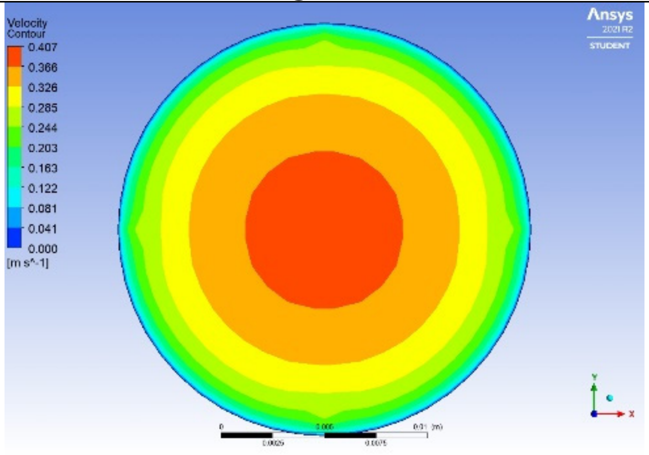

DW@ x/D = 20

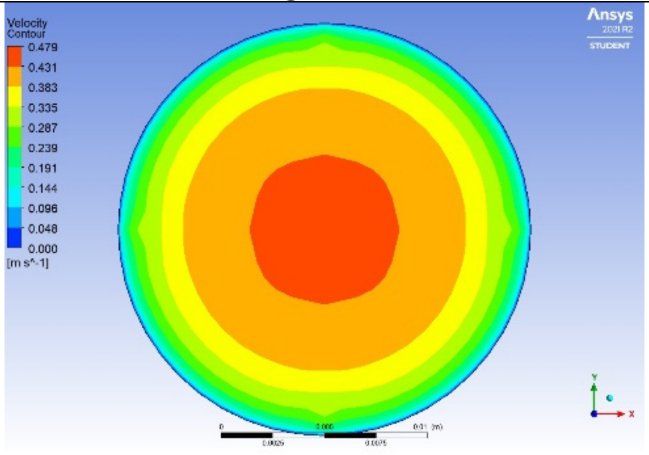

0.025%@ x/D = 5

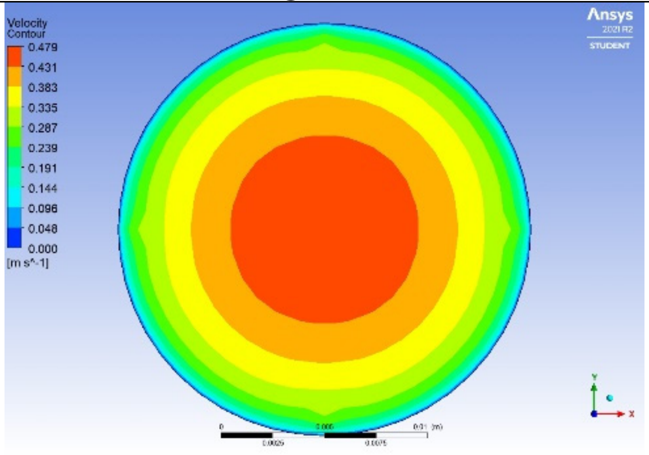

0.025%@ x/D = 10

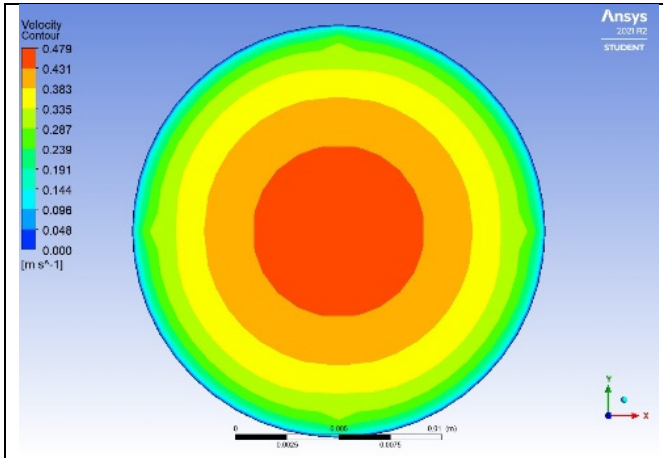

0.025%@ x/D = 15

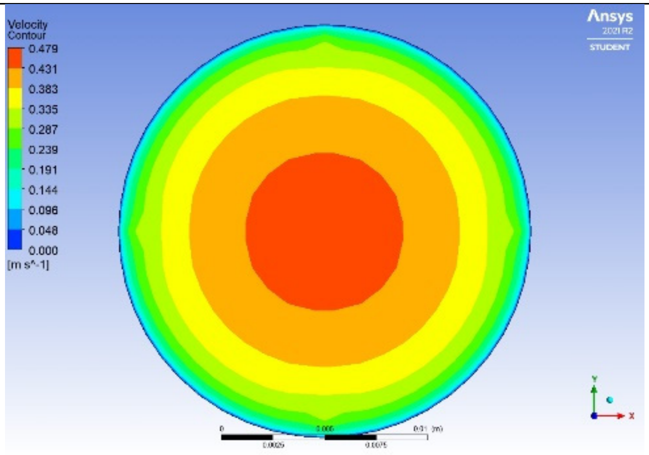

0.025%@ x/D = 20

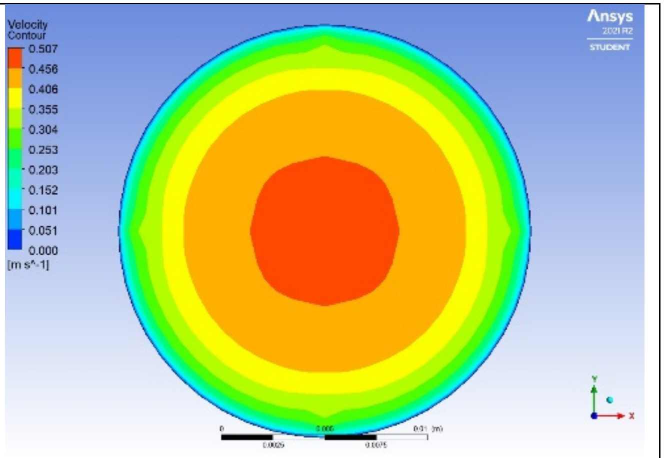

0.05%@ x/D = 5

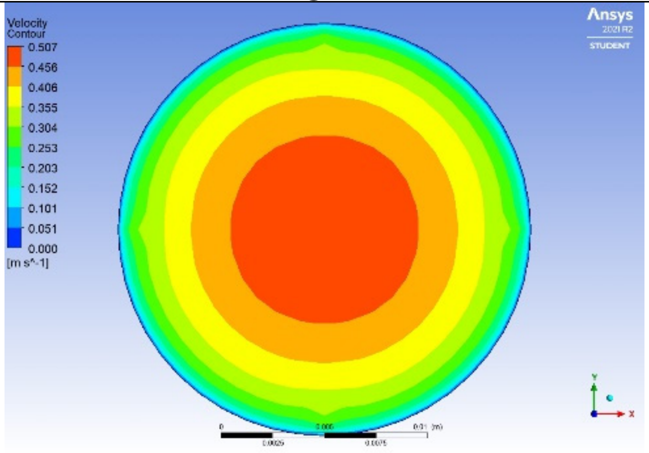

0.05%@ x/D = 10

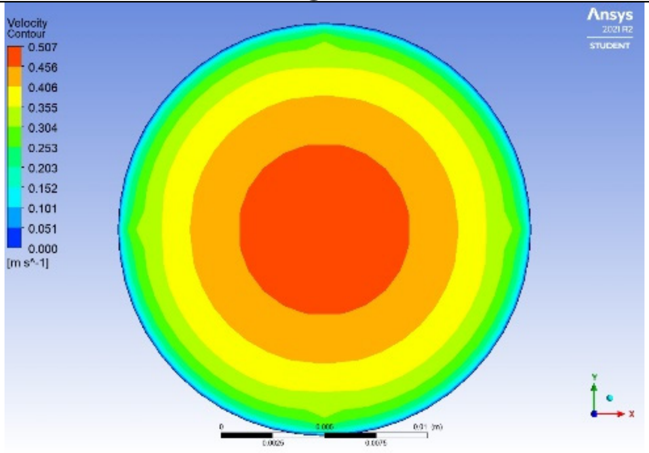

0.05%@ x/D = 15

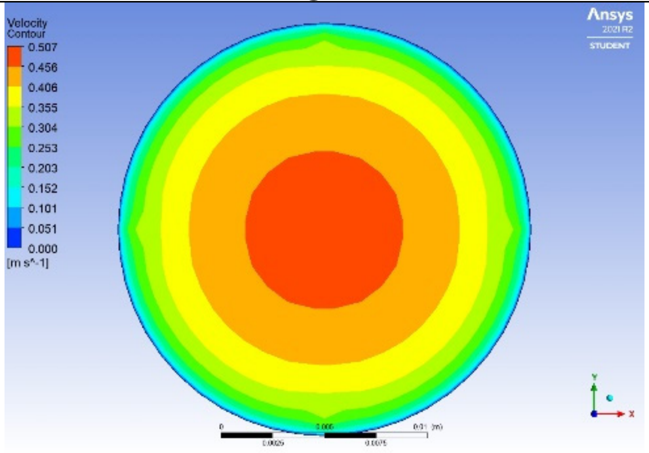

0.05%@ x/D = 20

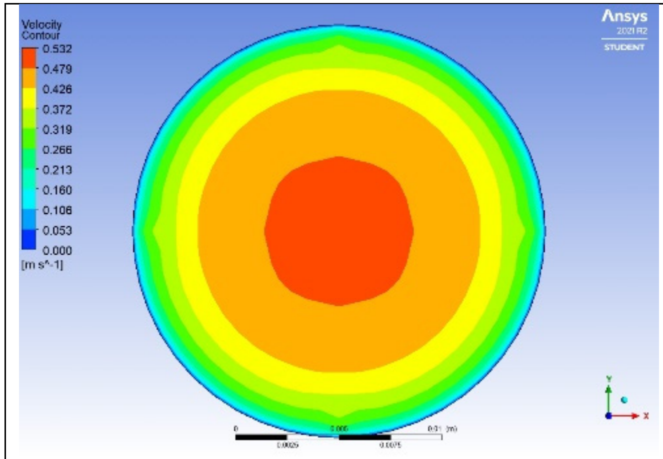

0.075%@ x/D = 5

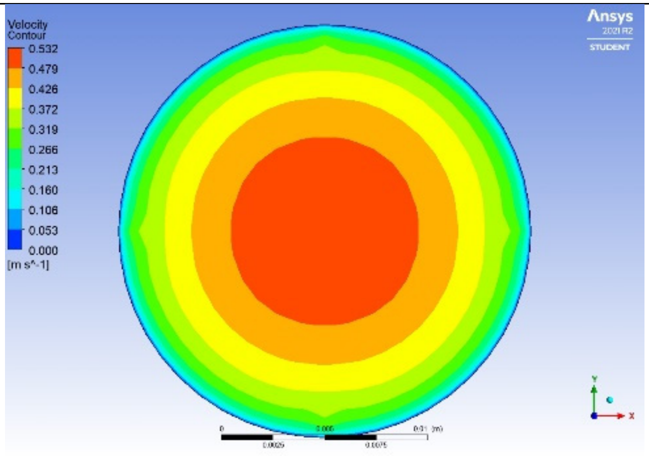

0.075%@ x/D = 10

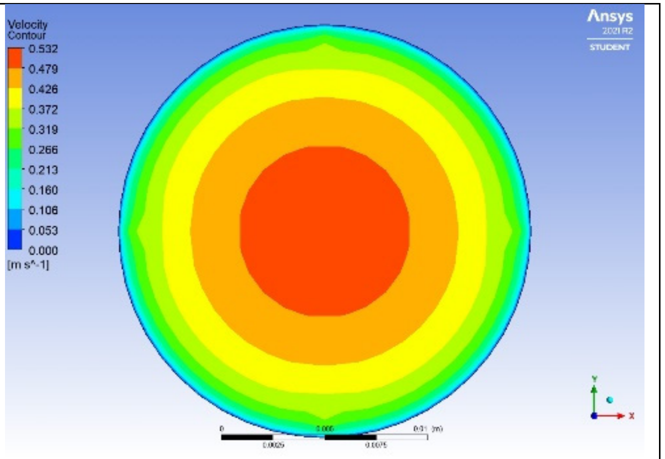

0.075%@ x/D = 15

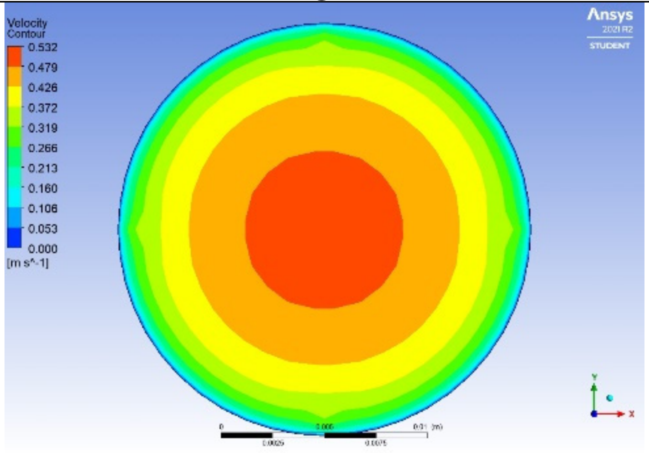

0.075%@ x/D = 20

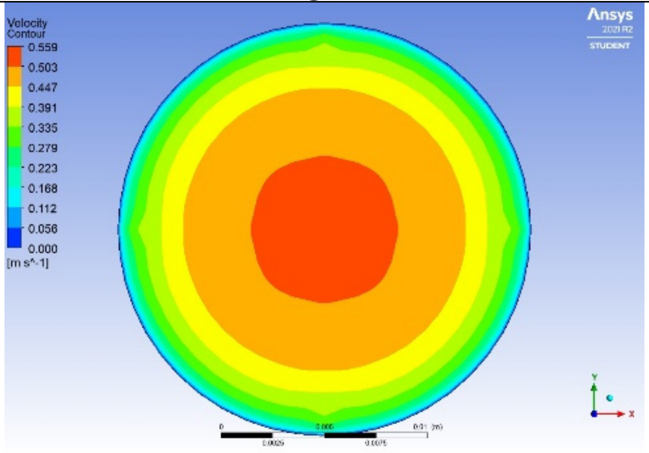

0.1%@ x/D = 5

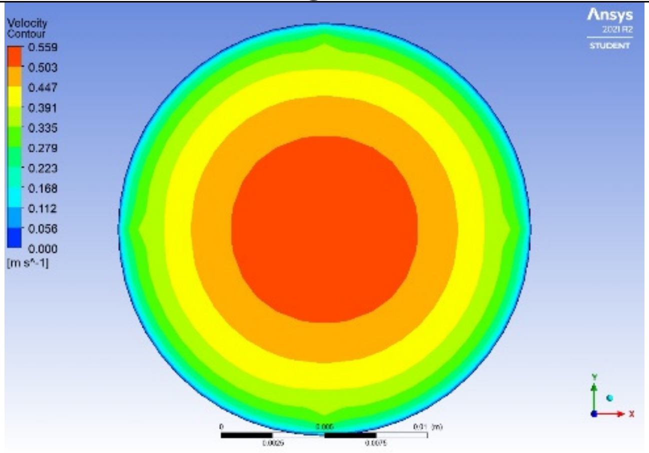

0.1%@ x/D = 10

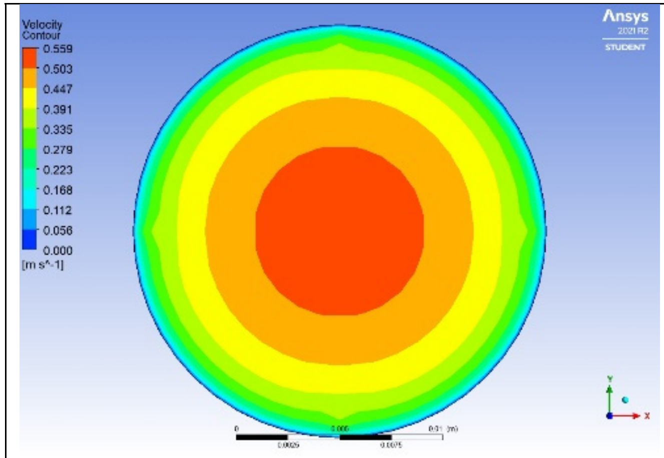

0.1%@ x/D = 15

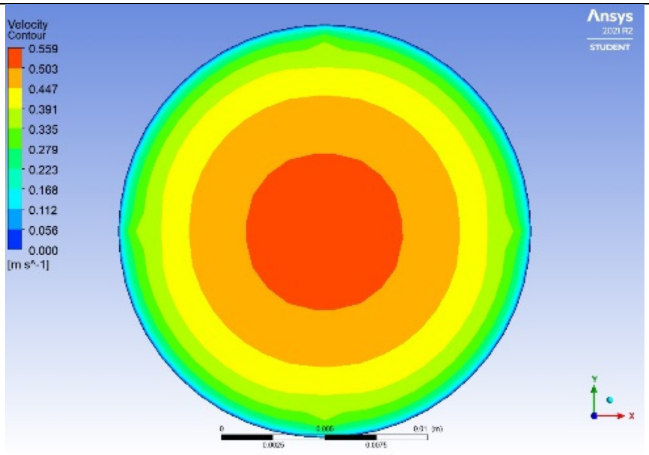

0.1%@ x/D = 20

Figure S2: Velocity contours of DW and different mass fractions of CF-GNPs at Re = 7,500 and different cross-sections.
